# Molecular basis of a redox switch: molecular dynamics simulations and surface plasmon resonance provide insight into reduced and oxidised angiotensinogen

**DOI:** 10.1042/BCJ20210476

**Published:** 2021-09-17

**Authors:** Jennifer M. Crowther, Letitia H. Gilmour, Benjamin T. Porebski, Sarah G. Heath, Neil R. Pattinson, Maurice C. Owen, Rayleen Fredericks, Ashley M. Buckle, Conan J. Fee, Christoph Göbl, Renwick C. J. Dobson

**Affiliations:** 1 Biomolecular Interaction Centre, University of Canterbury, Christchurch, New Zealand; 2 School of Biological Sciences, University of Canterbury, Christchurch, New Zealand; 3 Department of Biochemistry and Molecular Biology, Monash University, Melbourne, Victoria, Australia; 4 Medical Research Council Laboratory of Molecular Biology, Cambridge Biomedical Campus, Cambridge, U.K; 5 Centre for Free Radical Research, Department of Pathology, University of Otago, Christchurch, New Zealand; 6 Canterbury Scientific, Christchurch, New Zealand; 7 Department of Chemical and Process Engineering, University of Canterbury, Christchurch, New Zealand; 8 School of Product Design, University of Canterbury, Christchurch, New Zealand; 9 Department of Biochemistry and Molecular Biology, Bio21 Molecular Science and Biotechnology Institute, University of Melbourne, Melbourne, Victoria, Australia; 10 Maurice Wilkins Centre for Molecular Biodiscovery, University of Auckland, Auckland, New Zealand

## Abstract

Angiotensinogen fine-tunes the tightly controlled activity of the renin-angiotensin system by modulating the release of angiotensin peptides that control blood pressure. One mechanism by which this modulation is achieved is via angiotensinogen’s Cys18–Cys138 disulfide bond that acts as a redox switch. Molecular dynamics simulations of each redox state of angiotensinogen reveal subtle dynamic differences between the reduced and oxidised forms, particularly at the N-terminus. Surface plasmon resonance data demonstrate that the two redox forms of angiotensinogen display different binding kinetics to an immobilised anti-angiotensinogen monoclonal antibody. Mass spectrometry mapped the epitope for the antibody to the N-terminal region of angiotensinogen. We therefore provide evidence that the different redox forms of angiotensinogen can be detected by an antibody-based detection method.

## Introduction

The classical renin-angiotensin system (RAS) is a crucial hormone cascade responsible for the regulation of blood pressure in the body [1]. In the first and rate-limiting step of this pathway, the endopeptidase renin cleaves angiotensinogen to release the decapeptide angiotensin(Ang)-I. Ang-I is then cleaved by angiotensinogen-converting enzyme (ACE) to create Ang-II [2]. This octapeptide can be further hydrolysed to Ang-(1–7) by membrane-bound angiotensinogen-converting enzyme 2 (ACE-II), a receptor that has recently received attention as an entry point for different corona viruses, including SARS-COV and SARS-COV2 (Severe acute respiratory syndrome coronavirus) [3]. Ang-II activates angiotensin type I receptors (AT1R), leading to vasoconstriction and secretion of aldosterone. Aldosterone release results in sodium and water retention by the kidneys and a subsequent increase in blood pressure [4].

It has been well documented that angiotensinogen plays a fundamental role in fine-tuning the tightly controlled activity of the renin-angiotensin system and ultimately blood pressure itself, and has thus been associated with various hypertensive disorders and cardiovascular disease [5]. Typically circulating in the plasma at concentrations close to the *K_M_* of the renin-catalysed reaction [6] (~0.9 nmol/ml), angiotensinogen levels positively correlate with blood pressure [7,8]. In animal studies, blood pressure will decrease in response to an injection of antibodies specific to angiotensinogen [9], and conversely, an increase in blood pressure is observed following administration of an injection of angiotensinogen [10]. Further to this, it has been demonstrated that transgenic mice expressing rat angiotensinogen display elevated blood pressure [11]. A polymorphism within the human angiotensinogen gene, resulting in an amino acid substitution at position 235 (M235T), has been shown to be significantly associated with both increased plasma angiotensinogen levels and hypertension [12].

Angiotensinogen is further able to fine-tune the control of blood pressure via a redox switch mediated by its Cys18–Cys138 disulfide bond [13]. Cysteine residues play crucial roles in the structure and stability of proteins, and as such disulfide bond formation due to the surrounding redox environment can trigger changes in protein structure and activity [14,15]. Human angiotensinogen contains four cysteine residues but only Cys18 and Cys138, which form a disulfide bond under certain redox conditions, are conserved across species [12]. Angiotensinogen is oxidised in the endoplasmic reticulum and is subsequently secreted and reduced in the plasma, resulting in a consistent 60 : 40 ratio of the oxidised to reduced form in the circulation of healthy individuals, regardless of gender or age [13]. On incubation with renin in the presence of the (Pro)renin receptor, the oxidised form of angiotensinogen displays a four-fold increase in the catalytic release of Ang-I when compared with the reduced form of angiotensinogen [13]. The disulfide bond between Cys18 and Cys138 is crucial for constraining the N-terminus of angiotensinogen in order for the protein substrate to adopt a favourable conformation for reaction with renin [16,17].

Higher levels of the more active, oxidised form of angiotensinogen have been observed in hypertensive conditions, such as the pregnancy condition pre-eclampsia [13]. If we could reliably differentiate between the two redox forms of angiotensinogen this could represent a useful biomarker by which to monitor the progression of hypertensive disorders. With this in mind, we sought to probe the two redox states of angiotensinogen to gain a better understanding of the molecular structure of these intrinsically similar forms that differ only by the presence or absence of a disulfide bond. We hypothesised that, while the protein forms are likely to be structurally very similar, the dynamics of the two states may differ. Molecular dynamics simulations of each redox state of angiotensinogen reveal subtle dynamic differences between the reduced and oxidised forms, particularly at the N-terminus. To experimentally verify this *in silico* result, we demonstrate that the two redox forms of angiotensinogen display different binding kinetics to an immobilised anti-angiotensinogen monoclonal antibody by surface plasmon resonance and when we mapped the epitope for the antibody by mass spectrometry, we found that it binds to the N-terminal sequence of angiotensinogen. We therefore provide evidence that the different redox forms of angiotensinogen have altered dynamics and can be differentiated by an antibody-based detection method.

## Materials and methods

### Molecular dynamics simulations

Molecular dynamics simulations were carried out on human angiotensinogen (PDB entry: 2WXW), in both the reduced form (no disulfide bond between Cys18 and Cys138) and the oxidised form (Cys18 and Cys138 disulfide bonded). Missing loops and side chain residues were modelled using MODELLER [18]. All chain termini were capped with neutral groups (acetyl and methylamide).

Completed structures were solvated in a rectangular simulation box with sides measuring 99.82 Å × 39.05 Å × 86.47 Å, containing 12 sodium ions and 18 731 water molecules. Using the software Amber ver. 14, we modelled the ions and protein using the AMBER ff14SB force field, which is an extension of the AMBER ff99SB force field [19]. We modelled the water with the 3-particle TIP3P force field [20]. We applied SHAKE to all bonds involving hydrogen atoms [21]. We minimised the resulting structure to remove any clashes. We applied harmonic positional restraints of 10 kcal^−1^ mol^−1^ Å^2−1^ to the protein backbone atoms, kept the pressure at 1 atm and ramped up the temperature from 10 K to 310.15 K as a linear function of time over the course of 1.2 ns, using Berensden temperature and pressure control algorithms with relaxation times of 0.5 ps for both the barostat and thermostat [22]. We removed the restraints and performed a 5 ns simulation at constant isotropic pressure of 1 atm and temperature of 310.15 K to relax the system. An 8 Å cutoff radius was used for range-limited interactions with Particle Mesh Ewald electrostatics for long-range interactions [23].

The production simulation of oxidised and reduced angiotensinogen was carried out using NPT conditions. A Langevin thermostat was used to maintain the temperature at 310.15 K with a collision frequency of 2 ps, whilst Berensden pressure coupling was used to maintain the pressure at 1 atm with relaxation times of 2 ps. The simulation time step was 2 fs and snapshots were taken every 100 ps. Simulations were run in duplicate with Amber 14, using PMEMD on an Nvidia K20m GPU for 500 ns.

Graphs and plots were produced using Matplotlib [24]. Molecular graphics were prepared with PyMol version 2.3.4 [25] and Visual Molecular Dynamics (VMD) 1.9.2 [26].

### Recombinant protein expression and purification

The human angiotensinogen (AGT) gene (M235T variant) was a gift from Yahui Yan, University of Cambridge, U.K. The gene was cloned into two different bacterial vectors: a pET vector with an N-terminal hexa-histidine tag for purification and a pET-SUMO vector with an N-terminal hexa-histidine tag followed by a SUMO-tag to improve solubility. Either plasmid was transformed into *E*. *coli* BL21(DE3) and glycerol stocks of these cells harbouring the AGT expression plasmid were stored at −80°C. For expression, 15 ml of sterile Luria Burtani (LB) broth containing 50 μg/ml kanamycin was inoculated with 1 ml of the glycerol stock and incubated at 21°C overnight. The cells were then transferred into 950 ml of LB including kanamycin and grown to OD600 of 0.6 at 23°C. Protein expression was induced by addition of 1 mM isopropyl-β-D-1-thiogalactopyranoside (IPTG). Cells were incubated overnight at 19°C and harvested the following morning and pellets were stored at −20°C.

Cell pellets were thawed and re-suspended in purification buffer containing 20 mM HEPES, 110 mM potassium acetate, 20 mM imidazole and 5% (v/v) glycerol at pH 8.0. Lysis was performed by sonication on ice (UP200S, 0.5 s pulse per second for 12 min) followed by centrifugation at 15 000***g*** for 1 h at 4°C. The lysate was applied to a Ni-NTA gravity column (Qiagen) followed by wash steps with purification buffer according to the manufacturer’s protocol. The protein was eluted by purification buffer including 220 mM imidazole, then applied to a Superdex 200 PG (16/600) size exclusion column and eluted using purification buffer. The angiotensinogen peak fractions were pooled and purity was confirmed by SDS–PAGE. The sample was concentrated to 1 mg/ml using an Amicon 10 K centrifugal filter device and stored at −80°C. The 6xHis-SUMO-AGT protein was purified in the presence of 2 mM β-mercaptoethanol, whereas the 6×His-AGT was not. 6×His-AGT was used for antibody interaction studies with surface plasmon resonance and 6×His-SUMO-AGT was used for mass spectrometry-based epitope mapping experiments.

### Surface plasmon resonance

A ProteOn™ XPR36 Protein Interaction Array System using ProteOn™ GLC Sensor Chips and ProteOn™ Manager software (Bio-Rad Laboratories, Hercules, California, U.S.A.) was used to carry out the surface plasmon resonance studies. The antibody used was MAB3156 Clone 369439 Human Serpin A8, referred to as ‘anti-AGT mAb’, from R&D Systems (Minneapolis, Minnesota, U.S.A.). The antibody was immobilised as described below onto four channels at the same level of ligand density to serve as replicates. Mouse anti-human IgG Fc specific monoclonal IgG antibody clone 7QD (General Bioscience Corporation, Brisbane, California, U.S.A.), referred to as ‘anti-IgG mAb’, was immobilised onto one ligand channel to serve as a reference channel. All experiments were carried out at 25°C using filtered and sonicated PBST (phosphate buffered saline with Tween-20) as a running buffer.

### Antibody immobilisation

Ligands (anti-AGT mAb and anti-IgG mAb) were immobilised onto the sensor chip surface via amine coupling. The ligand channels were activated by a 150 μl injection of a 1 : 1 mixture of 0.4 M 1-ethyl-3-(3-dimethylaminopropyl)carbodiimide (EDAC) and 0.1 M *N*-hydroxysuccinamide (NHS) for 300 s at a flow rate of 30 μl/min to activate the carboxymethyl dextran on the sensor chip surface, to produce reactive succinimide esters. Ligand was then immobilised onto each channel by injecting 150 μl of 0.75 μg/ml antibody (in 10 mM sodium acetate buffer pH 4) for 300 s at a flow rate of 30 μl/min. Ligand immobilisation was directly followed by deactivation with 1 M ethanolamine HCl pH 8 at a flow rate of 30 μl/min for 300 s to deactivate any unreacted succinimide esters. Following two blank buffer injections, four stabilisation steps were performed in the ligand direction to stabilise the ligand surface and remove any weakly bound ligand molecules. Two 30 μl pulses of 10 mM sodium acetate pH 3 were followed by two 30 μl pulses of 10 mM glycine HCl pH 2, with each 18 s injection having a flow rate of 100 μl/min. Multiple blank buffer injections, first in the ligand direction and then in the analyte direction, were performed until a stable baseline was achieved across all channels.

### Interaction with reduced and oxidised angiotensinogen

Oxidised AGT was produced by purifying 6×His-AGT in the absence of reducing agent. Mass spectrometry analysis of NEM-derivatised oxidised AGT confirmed that the purified protein only has two accessible cysteine residues (Cys232 and Cys308) while reducing the protein with 50 mM DTT also makes Cys18 and Cys138 accessible for NEMylation (data not shown). Reduced AGT was prepared by overnight incubation with 2 mM DTT and PD-10 buffer exchange into PBST prior to the experiment. A dilution series of AGT with a dilution factor of two was prepared in PBST to give concentrations of 2 μM, 1 μM, 0.5 μM, 0.25 μM, 0.125 μM, and 0.0625 μM. A 409 μl injection of each AGT concentration, in descending order, was flowed simultaneously across separate channels on the chip in the analyte direction at a flow rate of 25 μl/min and allowed to associate with the anti-AGT mAb for a total contact time of 981 s. At the end of the injection, running buffer was flowed across all analyte channels for 1800 s (30 min) to allow dissociation. This was then followed with regeneration of the ligand surface — a 30 μl pulse of glycine HCl pH 2 for a total of 18 s at a flow rate of 100 μl/min to remove any non-dissociated analyte. At least four blank buffer injections were performed to allow the ligand to recover from regeneration and ensure a stable baseline was re-established. The order of the concentration series of AGT was then randomised and the interaction run was repeated such that each concentration was flowed across a different analyte channel to the previous interaction run. The procedure was carried out for both redox forms of AGT. Thus, a total of eight replicates for each of the reduced and oxidised forms of angiotensinogen at each concentration were measured (two replicates of four replicate ligand channels). The three highest concentrations of oxidised and reduced angiotensinogen were injected simultaneously across all ligand channels in the same run, first in an ordered fashion, and then in a randomised order, such that in each run oxidised angiotensinogen was flowed across three channels and reduced angiotensinogen was flowed across the other three channels.

All data sets were double referenced against both the anti-IgG mAb reference surface, and against buffer injections containing no protein. All data were processed using Scrubber 2 (Prot version, Biologic Software, Campbell, Australian Capital Territory, Australia) and fitted to a 1 : 1 Langmuir binding model using the ClampXP software to obtain kinetic association and dissociation constants [27]. Monte Carlo analysis was performed using ClampXP for each dataset by performing 50 independent rounds of model fitting with a maximum iteration of 25 cycles per fit, a variation of 1000% and added noise of 4 RU. Statistical analysis was carried out on the mean anti-AGT mAb surface kinetic constant values for oxidised and reduced AGT by performing independent sample *t*-tests (two-tailed, 95% confidence level) to test whether the kinetic values for the two forms differed significantly. Minitab 16 (Lead Technologies, Inc., Charlotte, North Carolina, U.S.A.) was used to perform the statistical analysis, and the data were checked for normality using the normality test prior to analysis.

### Epitope mapping

To produce sufficient quantities of human AGT, the protein was produced as a fusion with a SUMO tag to increase solubility, i.e. the 6×His-SUMO-AGT construct. Limited tryptic digestion was performed at a 50 : 1 substrate:trypsin (Promega) weight ratio at 37°C. Digestion was stopped by addition of 0.1% formic acid and samples were processed on the same day. Peptide design was guided by solubility analysis and sequences used for epitope confirmation were P1: ANAGKPKDPTFI (amino acid no. 24–35), P2: PAPIQAKTSPVD (36–47), P3: PVDEKALQDQ (45–54), P4: LVAAKLDTED (55–66), P5: EDKLRAAMVG (65–74), P6: NFLGFRIYGMHSE (78–90), P7: HTADRLQAIL (120–129) and P8: AILGVPWKDK (127–136). Solid-phase synthesised peptides were ordered from Mimotopes Pty Ltd (Australia) with purity >90%. The peptides were dissolved in double-distilled water to a concentration of 1 mM and 2 ml were used for dot blot analysis.

### HPLC/mass spectrometry

Tryptic peptides were analysed by LC–MS/MS using a Thermo Scientific Velos Pro ion trap mass spectrometer coupled to a Dionex UltiMate 3000 HPLC system with a 50 μl injection loop. Samples were stored on the auto-sampler tray at 5°C. A Jupiter 4 μm Proteo 90 Å column (150 × 2 mm, Phenomenex, Torrance, CA) at 40°C was used for chromatographic separation using water and 0.1% formic acid as Solvent A and acetonitrile and 0.1% formic acid as Solvent B. The column was equilibrated with 95% Solvent A and 5% Solvent B for 5 min then a linear gradient was run for 40 min to 50% Solvent A and 50% Solvent B to achieve separation. The column was flushed with 5% Solvent A and 95% Solvent B for 5 min and re-equilibrated to starting conditions for 5 min. An amount of 35 μg of digested protein was injected per sample and the flow rate was set at 0.2 ml/min. Nitrogen was used as the sheath gas and the temperature of the heated capillary was 275°C. Data analysis was performed with Thermo Xcalibur Qual Browser 2.2 SP1.48 (Thermo Fisher Scientific Inc., Waltham, MA). Identification and characterisation of peptides was performed using a double play method, first obtaining the full mass spectrum (*m*/*z* 300–1000) in positive-ion mode followed by fragmentation of the most abundant ions with helium gas collision-induced dissociation (fragmentation data not shown). A second identical sample was fractionated at 90 s intervals following separation by HPLC under the above conditions for subsequent antibody binding analysis. Both samples were analysed by UV detection at wavelengths 215, 254 and 280 nm using a Dionex UltiMate 3000 diode array detector to ensure their elution profiles matched.

### SDS–PAGE and western blotting

Samples were resolved by non-reducing 12% w/v SDS–PAGE alongside Precision Plus Protein Standards (Bio-Rad) as molecular weight markers. Resolved samples were either Coomassie stained or transferred to PVDF for western blotting with anti-AGT mAb (1 : 1000). The secondary antibody was horseradish peroxidase-conjugated goat anti-mouse (DAKO P044701). Antibody detection by chemiluminescence was performed using Amersham ECL Select Western blotting reagents and visualised using the UVItec Q9 Advanced Chemidoc. HPLC-separated fractions of digested protein were lyophilised and resuspended in 10 μl water. Dot-blot analysis was carried out by applying peptide solutions to PVDF membrane pre-soaked in methanol for immunoblotting with the above-mentioned antibodies.

## Results and discussion

### Reduced and oxidised angiotensinogen show a similar overall magnitude in dynamics, but subtle differences in motions across the structure, including at the N-terminus

**Figure 1:**
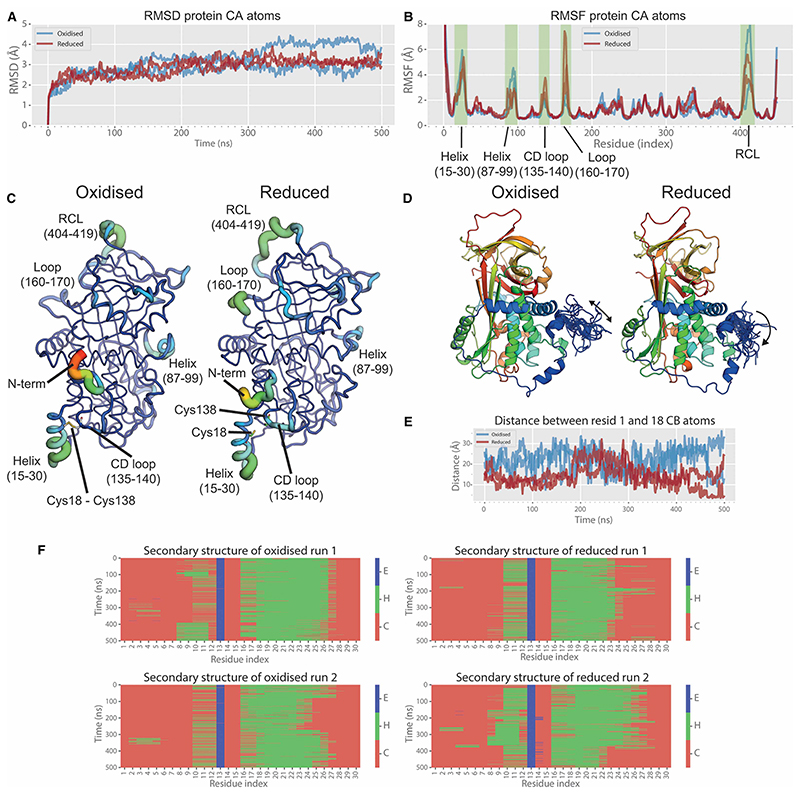
Molecular dynamics simulation of reduced and oxidised angiotensinogen. (**A**) RMSD plot and (**B**) RMSF plot of the Cα atoms of oxidised (blue) and reduced (red) human angiotensinogen over 500 ns. (**C**) Sausage plot displaying the RMSF values for both structures ranging from red (highest fluctuation) to blue (lowest fluctuation), dynamic regions are labelled. (**D**) Conformational sampling of the N-termini over the course of the simulations. (**E**) Distance between residue 1 and 18 over the course over the simulation. (**F**) Secondary structure analysis of the first thirty residues over the course of the simulation, E = extended beta-strand, H = helix, C = coil.

Molecular dynamics simulations were carried out on human angiotensinogen (PDB structure 2WXW) in the presence (oxidised) or absence (reduced) of the disulfide bond between Cys18 and Cys138 at 310.15 K in duplicate for simulation trajectories of 500 ns. Initial analysis was carried out by calculating the root mean square deviation (RMSD) and root mean square fluctuation (RMSF) across the trajectories for both reduced and oxidised angiotensinogen. The RMSD plot (deviations of the Cα atoms from the initial energy minimised structure through time) reveals similar deviation patterns for both oxidised and reduced angiotensinogen (Figure 1A). We observe a rough equilibration by 100 ns, with a slow and gradual increase in deviation from the starting coordinates over the next 400 ns. We note that the reduced simulations show a much more convergent simulation trajectory, while the oxidised simulations deviate by ~1 Å from ~300 ns. It is unclear from the RMSD plot alone, what the significance of this is.

Inspection of the RMSF plot (Figure 1B), which highlights the deviation of each residue over the course of the simulation, reveals that there are distinct differences in the dynamics of key regions of reduced and oxidised angiotensinogen. As shown in Figure 1B,C, we observe differences in the fluctuations of the N-terminus, a helix (87– 99), the CD loop (135–140, loop connecting helices C and D) and loops 160–170 and 404–419 (reactive centre loop). Interestingly the N-terminus of the oxidised form fluctuates to a higher degree compared with the reduced form; this may seem counter-intuitive given the presence of the disulfide bond in the oxidised form, however, it appears that the lack of a disulfide bond in the reduced form alters the conformational sampling of the N-terminus, with simulations showing a preference for the N-terminus to embed itself in the space where the disulfide bond would normally be (Figure 1D,E). Although the RMSF values of the A2 helices (residues 15–30) are very similar between oxidised and reduced angiotensinogen, we observed an overall reduction in secondary structure of this helix in the reduced form (Figure 1F — see the shift from green (helices) to red (random coil) for residues 22 onwards between oxidised (left) and reduced (right)). This is likely due to the lack of restraint from the Cys18–Cys138 disulfide bond, which in turn has a knock-on effect of increasing the flexibility of the CD loop.

### An SPR assay is able to distinguish between reduced and oxidised angiotensinogen

We next studied the binding properties of a commercially available monoclonal antibody known to bind angio- tensinogen (anti-AGT mAb) using a surface plasmon resonance (SPR) based method. This method provides the unique capability to measure both the kinetic association (*k*_on_) and dissociation (*k*_off_) rate constants and therefore allows detailed insights into the binding kinetics. We expressed recombinant angiotensinogen carrying an N-terminal hexa-histidine tag in *E*. *coli* and purified the protein by nickel affinity and size exclusion chromatography.

**Figure 2:**
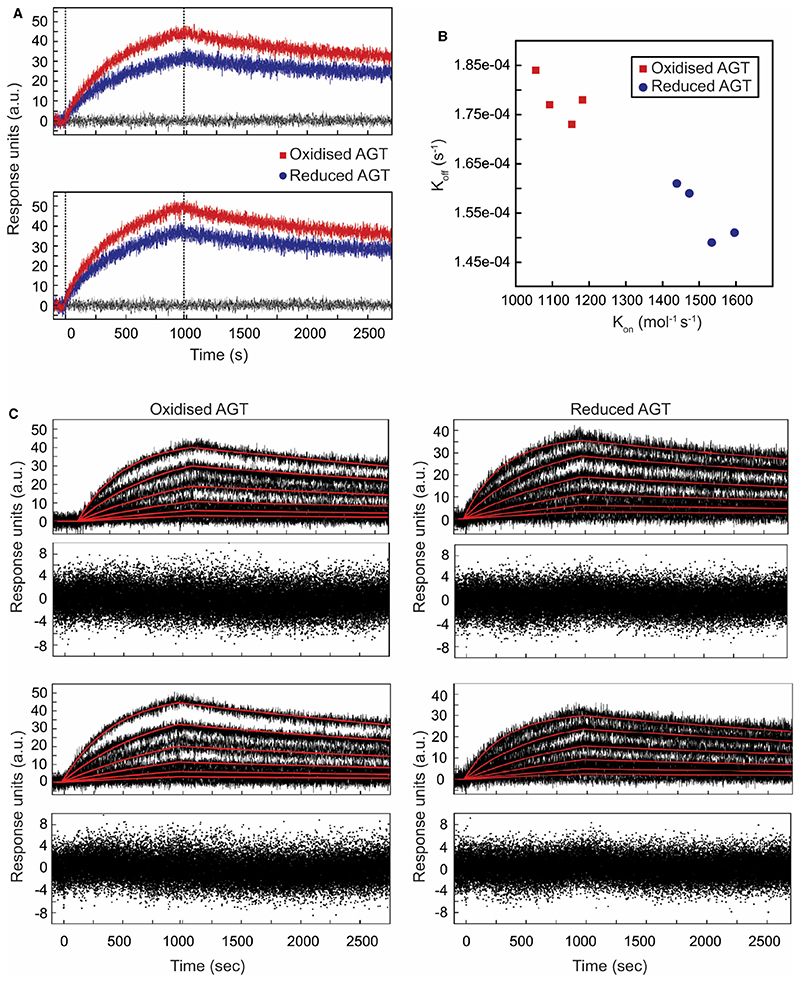
SPR analysis of reduced and oxidised AGT. (**A**) Examples of overlapping sensorgrams from binding of reduced (blue) and oxidised (red) AGT to immobilised anti-AGT mAb (buffer reference in black). (**B**) Plot of *k*_off_ vs. *k*_on_ for reduced and oxidised AGT determined by fitting of dose dependence data with a 1:1 binding model. (**C**) Examples of biosensor dose dependence curves as in (**A**) including data fits (red).

We performed a multiplex approach for data collection using repeat analyte injections. Analysis showed that the results obtained were accurate and reproducible. The anti-AGT mAb was immobilised at the same concentration on four activated ligand channels with a further channel immobilised with an anti-IgG mAb at the same concentration to serve as a reference surface. Examples of the binding responses (including replicates) obtained with oxidised and reduced angiotensinogen are displayed in Figure 2A.

The sensorgrams, although noisy, show concentration-dependent responses as a result of reduced or oxidised angiotensinogen binding to the anti-AGT mAb surface (Figure 2A). Replicates of each analyte concentration overlay well and there are only subtle differences in the magnitude of responses between ligand channels, indicating that these results are reproducible. Although ligand saturation was not reached for the highest concentration, each set of sensorgrams shows visible curvature in the association phase and decay in the dissociation phase, allowing for detailed kinetic analysis.

To demonstrate the reproducibility of the results, the data from each anti-AGT mAb ligand channel was analysed and fitted to a kinetic model separately and independently of the other ligand channels. All binding data from reduced and oxidised angiotensinogen fitted well to a 1 : 1 Langmuir binding model. As shown in Figure 2B, both the *k*_on_ and *k*_off_ rate constants were different for the two redox forms, with oxidised angiotensinogen displaying a slower association rate and faster dissociation rate than reduced angiotensinogen (average *k*_on_ of 1121 M^−1^ s^−1^ vs. 1510 M^−1^ s^−1^ and average *k*_off_ of 1.78 × 10^−4^ s^−1^ vs. 1.55 × 10^−4^ s^−1^, respectively). These differences in the association and dissociation rate constants led to different dissociation constants (*K_d_* = *k*_off_/ *k*_on_) for the angiotensinogen-antibody complex, depending on the redox state of the protein. Oxidised angiotensinogen was determined to have a mean *K_d_* value of 159 nM while reduced angiotensinogen had a mean *K_d_* value of 103 nM and therefore binds more tightly.

Statistical analysis revealed that the differences in the *k*_on_, *k*_off_, and *K_d_* values between reduced and oxidised angiotensinogen are statistically significant, having *P*-values of <0.0001 (Table 1). Furthermore, each data set was subjected to Monte Carlo analysis. When varying starting values up to 1000% and applying 50 cycles of Monte Carlo fits and addition of random short-term noise, all results converged on the same set of *k*_on_ and *k*_off_ values, strongly supporting the validity of the kinetic model fitting to each data set. The residuals were randomly distributed across the baseline with an amplitude of ±4 RU, similar to the noise present on the sensorgrams (Figure 2C). The kinetic constants and standard deviations resulting from the best fit to the data for each surface, for both reduced and oxidised angiotensinogen, are summarised in Table 1. The standard deviations from the fitting of the kinetic parameters are small, indicating that the rate constants for each data set are well defined.

Overall, these results demonstrate that by using the antibody-based SPR assay developed here, recombinant non-glycosylated reduced and oxidised angiotensinogen can be experimentally differentiated in a reliable and reproducible fashion. The anti-AGT mAb antibody surface has a higher affinity for the reduced form of angiotensinogen when compared with the oxidised form, due to differences in both the association and dissociation rates of the two redox states of angiotensinogen when interacting with the immobilised antibody. Although we used N-terminal His-tags to aid purification, in our SPR binding studies the reduced and oxidised forms of angiotensinogen carry the exact same N-terminal His-tag and therefore the different binding affinities are due to the redox state and not the tag.

### Mass spectrometry analysis maps the anti-AGT mAb epitope to a region predicted to have altered dynamics upon oxidation

**Figure 3:**
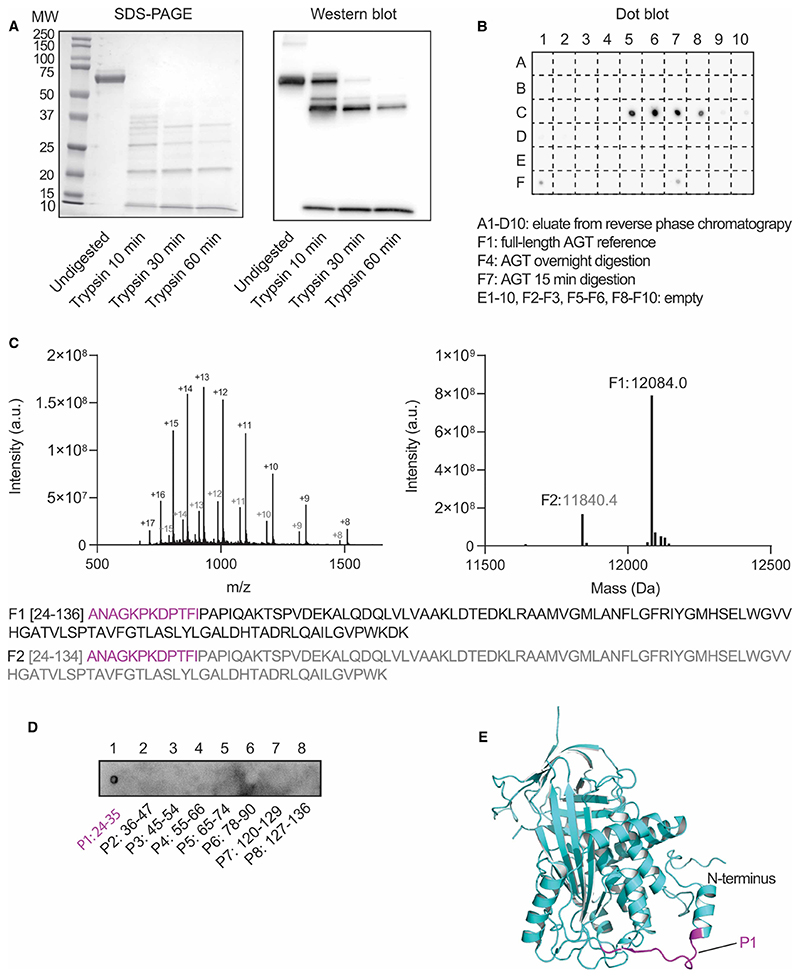
Epitope mapping of the anti-AGT mAb. (**A**) Left, SDS–PAGE of 6xHis-SUMO-AGT (undigested) and after trypsin digestion for 10, 30 and 60 min. Right, Western blot of the same samples visualised by blotting with anti-AGT mAb. Several bands are identified that include the epitope, including the full length protein at 63 kDa, two bands at ~40 kDa that represent trypsin cleavage at sites distant to the epitope, and a small band at ~12 kDa which represents cleavage that has occurred close to the epitope. (**B**) Dot-blot of a 15 min trypsin digested sample and chromatographic separation, followed by probing with anti-AGT mAb, control samples are included as labelled. (**C**) Mass spectrometry analysis of the same eluate as in **B** highlights the presence of two N-terminal cleavage products (F1, F2). (**D**) Dot-blot of eight peptide fragments covering the sequence of F1. (**E**) Structure of full-length AGT (PDB ID: 2WXW) highlighting the location of P1 in magenta.

Next, we determined the binding region of the anti-AGT mAb to angiotensinogen using mass spectrometrybased epitope mapping [28]. For this, we applied a limited digestion approach followed by western blotting in order to identify if a linear epitope is recognised. The protein was trypsin digested for different amounts of time, followed by SDS–PAGE and western blotting using the anti-AGT mAb. Several peptide fragments of different sizes showed binding, indicating the presence of a linear motif (Figure 3A). Only short proteolysis times resulted in antibody recognition while overnight incubation completely degraded the epitope (Figure 3B, position F4).

To identify the binding site, we performed a 15 min limited digestion step and applied one half of the sample to a hydrophobic interaction column allowing separation of the different cleavage products. The eluate was directly coupled to an ion-spray mass spectrometer to yield the mass of the peptides. The other half of the sample was applied to the same column and fractionated at 90 s intervals resulting in 30 ml aliquots, followed by dot blot analysis with anti-AGT mAb (Figure 3B). This parallel approach enabled identification of the peptide fragments present in each fraction and their potential binding to the antibody. We found that two peptides of ~12 kDa corresponding to residues 24–134 and 24–136 from the N-terminal region of angiotensinogen were recognised by the antibody (Figure 3C).

To narrow down the epitope to a shorter region, we designed 8 linear, short peptides covering this region (residues 24–136) and generated them by solid-phase synthesis. A dot blot of these fragments showed exclusive binding of the antibody to peptide number 1, which covers the sequence from amino acids 24 to 35 (Figure 3D). This analysis shows that the epitope of anti-AGT mAb is linear and is located in the N-terminal region near helix A2 (residues 15–30). Given that we see differences in this region in the molecular dynamics simulations, specifically a loss of secondary structure in the oxidised form, this could explain why we see differential binding of the anti-AGT mAb between the reduced and oxidised states of angiotensinogen.

## Conclusion

Angiotensinogen is a key regulator of the renin-angiotensin system, exerting its influence on blood pressure through alteration of protein levels, or protein activity via a redox switch. Here, we investigated the dynamic differences between the redox states of angiotensinogen.

Molecular dynamics simulations indicate that the dynamics within certain regions of angiotensinogen change as a function of the redox state. Understandably, the presence of a disulfide bond between Cys18 and Cys138 in the oxidised form constrains the movements of the neighbouring A2 helix (15–30) and the CD loop (135–140). The N-terminus itself (proximal to the A2 helix) samples an increased conformational ensemble in the oxidised form, as when the disulfide bond is reduced, the N-terminus appears to prefer binding in the cavity vacated by the bond. The protease renin is responsible for the cleavage of the N-terminal decapeptide Ang-I, and the angiotensinogen-renin complex is stabilised by the N-terminal region, helices A and C, and the CD loop [13,17]. In line with our observation of major differences in these regions, renin shows a four-fold higher affinity for the oxidised form of angiotensinogen [13].

We further investigated the question of whether a single, monoclonal antibody can differentiate between the two subtly different redox forms of angiotensinogen. For this we turned to surface plasmon resonance, a method that can provide binding insights through the elucidation of association and dissociation rate constants. We found that the two redox states of angiotensinogen exhibit different forms of binding to the same antibody.

The reduced form shows higher *k*_on_ and lower *k*_off_ rates, resulting in a slightly lower *K_d_*, indicating tighter binding compared with the oxidised form. These results suggest that the monoclonal antibody binds to an epitope that is influenced by the redox state of angiotensinogen, but is still accessible in both redox forms.

We therefore characterised the binding site of the monoclonal antibody. A combination of limited digestion, peptide separation using reverse-phase HPLC and western blotting methods identified a unique, N-terminal binding site of the antibody. After initial identification of the region between residues 24–136, the binding to different short peptides covering this sequence showed antibody interactions to peptide 1 only, consisting of amino acids 24–35. This region is located in the flexible N-terminus that according to the crystal structure forms an extended, linear motif easily accessible on the surface of the protein.

Our molecular dynamics simulations reveal that this motif, which includes part of the helix between residues 15–30, is in a highly dynamic region that is strongly impacted by the redox state. While only subtle differences in overall RMSF were observed in this region between the reduced and oxidised forms, the C-terminal region of the helix (residues 22–30) shows consistent loss of alpha-helical structure in the reduced version, which likely contributes to the altered binding affinity of the antibody.

In conclusion, we have observed an experimentally detectable difference in antibody binding, when assessed by SPR, of the two redox forms of angiotensinogen. This could be utilised in a diagnostic setting as a means to assess the progression of, or even pre-diagnose, hypertensive disorders, such as pre-eclampsia.

## Figures and Tables

**Table 1:** SPR fitting parameters of the interaction of anti-AGT mAb with AGT, a 1 : 1 binding model was applied to fit the data

AGT binding parameters			
	*k*_on_ (M^−1^ s^−1^)	*k*_off_ (s^−1^)	*K*_d_ (nM)
Oxidised AGT	1121 ± 4	(1.78 ± 0.01) × 10^−4^	159 ± 1
Reduced AGT	1540 ± 6	(1.55 ± 0.01) × 10^−4^	103.1 ± 0.9
Independent Samples *t*-test (P-value)	<0.0001	<0.0001	<0.0001
*t*-value	−107.89	32.53	83.10
Degrees of freedom	5	5	5
**Individual measurements, oxidised AGT**			
**Anti-AGT mAb surface**	***k*_on_ (M^−1^ s^−1^)**	***k*_off_ (s^−1^)**	***K*_d_ (nM)**
Surface 1	1182 ± 4	(1.78 ± 0.01) × 10^−4^	150 ± 1
Surface 2	1153 ± 4	(1.73 ± 0.01) × 10^−4^	150 ± 1
Surface 3	1055 ± 4	(1.84 ± 0.01) × 10^−4^	174 ± 1
Surface 4	1093 ± 3	(1.77 ± 0.01) × 10^−4^	162 ± 1
**Individual measurements, reduced AGT**			
**Anti-AGT mAb surface**	***k*_on_** **(M^−1^ s^−1^)**	***k*_off_** **(s^−1^)**	***K*_d_** **(nM)**
Surface 1	1439 ± 6	(1.61 ± 1) × 10^−4^	112 ± 1
Surface 2	1534 ± 6	(1.49 ± 1) × 10^−4^	97.3 ± 0.9
Surface 3	1596 ± 6	(1.51 ± 1) × 10^−4^	94.8 ± 0.9
Surface 4	1473 ± 5	(1.59 ± 1) × 10^−4^	108.1 ± 0.8

## Data Availability

All relevant data are included within the manuscript.

## Abbreviations

ACE: angiotensinogen-converting enzyme
AGT: angiotensinogen
RMSD: root mean square deviation
RMSF: root mean square fluctuation
SPR: surface plasmon resonance
